# Neuropsychological Assessment of Cognitive Impairment in Kidney Transplantation (NAsKiT) and its related risk factors: a study protocol

**DOI:** 10.1007/s40620-022-01376-z

**Published:** 2022-06-28

**Authors:** Hristos Karakizlis, Johanna M. Doerr, Anna Becker, Christian Nahrgang, Lucy Rainer, Ingolf Askevold, Juliane Liese, Winfried Padberg, Mostafa Aly, Rolf Weimer, Martin Juenemann

**Affiliations:** 1 Division of Nephrology and Kidney Transplantation, Department of Internal Medicine II, University Hospital Giessen and Marburg, Justus-Liebig-University Giessen, Klinikstrasse 33, 35392 Giessen, Germany; 2 Department of Neurology, University Hospital Giessen and Marburg, Justus-Liebig-University of Giessen, Giessen, Germany; 3 Department of General, Visceral and Thoracic Surgery, Justus-Liebig-University of Giessen, Giessen, Germany; 4 Transplantation Immunology, Institute of Immunology, University Hospital Heidelberg, Im Neuenheimer Feld 305, 69120 Heidelberg, Germany; 5 Nephrology Unit, Internal Medicine Department, Assiut University, Assiut, Egypt

**Keywords:** Kidney transplantation, Cognitive performance, Neuropsychological assessment, Depression, Cognitive profile, Chronic kidney disease

## Abstract

**Background:**

Association of cognitive impairment with chronic kidney disease has been reported over the last decade. Individuals show better cognitive performance after kidney transplantation than individuals on dialysis but are more likely to be affected by cognitive impairment than age-matched comparison groups. Better knowledge of the prevalence as well as course and profile of cognitive impairment is important for the design of future studies assessing the clinical impact of cognitive impairment and developing management strategies. The goal of our study is to examine the extent of cognitive impairment before and after transplantation and to derive a distinct profile of cognitive function using standard neurocognitive tests. Furthermore, we aim to assess whether transplantation per se leads to an improvement in cognitive performance.

**Methods:**

We are conducting a prospective single-center cohort study involving 100 kidney transplant individuals. Individuals who are wait-listed to receive a kidney transplantation or have already received one will be included in this study. Individuals will undergo a battery of detailed neurocognitive tests at baseline (in part before surgery), and then 3 and 12 months afterwards. Furthermore, the enrolled patients will complete a validated German version of the Cognitive Failure Questionnaire for self-assessment (s-CFQ) as well as the Hospital Anxiety and Depression Scale -Deutsche (HADS-D), a self-report screening instrument with two scales that capture anxiety and depression. In addition, a hair sample will be taken at each measurement time point for the determination of hair cortisol levels as a parameter for the cumulative hypothalamic-pituitary-adrenocortical axis activity over the previous three months. The primary outcome measure will be (a) the effect of kidney transplantation on the cognitive performance up to 12 months after transplantation and (b) the course of cognitive performance following kidney transplantation over time.

**Discussion:**

The results of our study have potentially important implications for the prevention and treatment of cognitive impairment in kidney transplant individuals. By increasing our knowledge of the neurocognitive profile and assigning the corresponding deficits, it might be possible to create an individualized training program to positively impact cognitive deficits in kidney transplant patients.

## Introduction

Kidney transplantation is the preferred therapy for individuals with end-stage renal disease (ESRD). To date, efforts to improve cardiovascular and metabolic parameters after transplantation have been made, but the cognitive aspects of chronic kidney disease (CKD) have been relatively overlooked. Association of cognitive impairment with CKD has been reported over the last decade [1–5]. Several studies suggested a prevalence of cognitive impairment in up to 80% of individuals with CKD [1, 2, 5–9]. It has been demonstrated that individuals showed better cognitive performance after kidney transplantation than dialysis individuals [10] but these individuals are also significantly more likely to be affected by cognitive impairment or dementia than age-matched comparison groups [11–13]. Additionally, kidney transplant recipients with cognitive impairment are also affected by increased mortality [14, 15]. However, the actual prevalence of cognitive impairment in transplant recipients (from deceased and living donor) is unknown. The development of cognitive functioning and the detailed degree (i.e., which cognitive domains are particularly affected) has not yet been well studied in kidney transplant individuals. Previous studies in individuals with end-stage renal disease have shown that executive functions are particularly affected [5].

Cognitive impairments are likely to imply a high individual loss of quality of life and early restriction of self-determination and of therapy adherence, which is highly necessary for treatment. Early detection is of paramount significance so as to take preventive action, assess illness-related grief, and to avoid misunderstandings during medical care [16].

Better knowledge of the prevalence as well as of the course and profile of cognitive impairment is important for designing future studies, which will assess the clinical impact of cognitive impairment and develop management strategies. A limited number of studies have concentrated on the effects of kidney transplantation on cognitive function with divergent results [13].

The goal of our study is to examine (a) the extent of cognitive impairment, (b) the course of cognitive performance in kidney transplant individuals and (c) the profile of cognitive impairment (i.e., if a certain cognitive domain is more affected than others). Furthermore, we explore the impact and temporal course of variables with a potential influence on cognitive performance as secondary research questions. In this context, variables related to stress (subjective stress, depression, and long-term activity of the hypothalamic pituitary adrenal axis (HPA)) are of special interest to us.

## Materials and methods

### Study design and enrollment

We are conducting a prospective single-center cohort study at the University Hospital Giessen and Marburg, Giessen, Germany. It complies with the Declaration of Helsinki and has been approved by the ethics committee of the Justus Liebig University Giessen (ref 195/20). Written informed consent will be signed by the individuals and by the investigator prior to the patient´s enrollment.

Our study team consists of members of the nephrology, kidney transplantation, neurology and neuropsychology departments.

### Inclusion and exclusion criteria

Individuals who are scheduled to receive a kidney transplantation (immediate—deceased kidney donor, or within the following 2 weeks—living kidney donors) or have already received a kidney transplantation in the past will be included in this study. Due to the use of a standardized psychological assessment, individuals have to be native German speakers and at least 18 years of age. individuals under the legal supervision of a caregiver and with preexisting psychiatric disorders will be excluded from the study.

The study is divided into two parts (Table 1).

**Table 1:** Trial schedule of enrollment and assessments

		Enrollment		Post-allocation	
Study Period, Part A					
Time point		*t*_−1_	*t*_1_	*t*_2_	*t*_3_
Enrollment					
Eligibility screen		X			
Informed consent		X			
Allocation		X			
Interventions					
Kidney transplantation			X		
Assessments					
ROCFT		X		X	X
VLMT		X		X	X
TMT-A		X		X	X
WMS-R-numbers forward		X		X	X
TMT-B		X		X	X
WMS-R-numbers backwards		X		X	X
RWT		X		X	X
Study period, Part B					
Time point	*t*_−2_		*t*_−1_	*t*_1_	*t*_2_
Enrollment					
Eligibility screen			X		
Informed consent			X		
Allocation			X		
Interventions					
Kidney transplantation	X				
Assessments					
ROCFT			X	X	X
VLMT			X	X	X
TMT-A			X	X	X
WMS-R-numbers forward			X	X	X
TMT-B			X	X	X
WMS-R-numbers backwards			X	X	X
RWT			X	X	X

Part A:

All included individuals undergo a detailed neurocognitive test battery on the day of transplantation (deceased kidney donation), or within 14 days before transplantation (living kidney donation). The neurocognitive test battery will be repeated after 3 and 12 months.

For individuals on the waiting list for deceased donor kidney transplantation we aspire to also carry out neurocognitive tests during standard visits to the hospital. Standard visits are typically several years apart and the date of transplantation is unforeseeable. Therefore, we expect not to be able to gather respective data for the majority of our participants and rather, will use it descriptively and for qualitative analyses.

Part B:

All included individuals who have already undergone transplantation and are in outpatient follow-up will undergo the same neurocognitive test battery as individuals in part A at baseline, after 3 months, and after 12 months.

### Neuropsychological assessment

A battery of standardized cognitive tests will be performed at different timepoints (Table 1). Parallel test forms will be used at follow-up to account for learning effects. The order in which the parallel test forms are presented will be counterbalanced so that each parallel test form wil be administered with the same frequency at each test time point.

#### The cognitive test battery

We first administer the Mini-Mental Status Examination (MMSE) [17] as a neurocognitive screening test, together with a test battery consisting of five tests, assessing the cognitive domains of selective attention, verbal and visual memory with short-delay and long-delay memory conditions, verbal working memory, word fluency and symbol processing. Raw scores are transformed into z-scores adjusted for age, and if available sex, as well as years of education.

The individual’s degree of impairment will be stratified using the algorithm adopted by Murray and colleagues [1] based on the Mayo criteria for mild cognitive impairment (MCI) [18] and the Diagnostic and Statistical Manual, fifth Edition, criteria for major neurocognitive impairment as approximate guidelines [19].

Individuals will be classified depending on their extent of impairment and the number of affected domains as.

1. Unimpaired: performance better than 1.5 standard deviations (SD) below the norm sample in any test considered for the classification.
2. Mildly impaired: mild deficits (scores 1.50 to 1.99 SD below the norm sample) in only one domain.
3. moderately impaired: deficits in two domains or a severe deficit (2.0 SD below the norm Sample) in one domain.
4. Severely impaired: deficits in at least two domains.

The domains of executive functions (semantic and phonemic fluency, working memory, and mental flexibility), verbal memory (immediate and delayed recall, recognition), visual memory (delayed recall), and attention (symbol processing speed) are considered in the classification.

For this purpose, we will use the following validated tests (Table 2):

**Table 2:** The different neurocognitive tests and their affiliation to a particular cognitive domain

Cognitive domain	Test
Visual memory	ROCFT
Verbal memory	VLMT
Attention	TMT A WMS-R–numbers forward
Executive function	TMT B WMS-R–numbers backwards RWT

### Verbal learning and memory test

To assess verbal memory, the “verbaler Lern- und Merkfähigkeitstest” (VLMT) [20], a modified German version of the Rey Auditory Verbal Learning Test [21] will be administered. This test can be used to evaluate short-term memory, learning, episodic memory and verbal discriminability. First, a list of 15 words is read to the patient by the investigator. The direct retrieval by the patient is scored as short-term memory performance. Second, the patient has to learn the word list in five learning trials. The sum of the recalled words represents a learning parameter. Third, a second word list with new words is presented verbally, and recalled only once for interference. After this, the learned words on the first word list have to be recalled. This is used as a measurement of a short-delayed function of verbal episodic memory. A second verbal episodic memory measurement is performed 20 min later (long delay). Finally, the verbal recognition ability is assessed by discriminating between already learned and new words. Between the short-delayed verbal episodic memory trial and the long-delayed verbal episodic memory trial, nonverbal cognitive tests are performed to avoid the potential effect of interfering words not included in the learned wordlist. Three parallel versions of this test are available and are implemented during baseline and follow-ups in alternating order.

### Rey-Osterrieth Complex Figure Test

In the Rey-Osterrieth Complex Figure Test (ROCFT) [22], the subject is first asked to copy a complex figure (visuo-construction/action planning). After about 30 min, the subject is then asked to draw the figure again from memory (visual memory).

### Trail Making Test

Selective attention and cognitive flexibility are examined using the Trail Making Test (TMT) [23]. It consists of subtests A and B. In TMT-A, the patient has to link numbers in ascending order as quickly as possible with a pencil (visual scanning and basal psychomotor speed, selective attention) on a test sheet. TMT-B additionally requires the ability of cognitive flexibility by switching between letters and numbers. The quotient formed from the processing time of subtest B and subtest A (B/A) can be used to represent a measure of cognitive switching ability (serial cognitive flexibility, executive function) independently of any psychomotor slowing.

### Wechsler Memory Scale (subtest digit span)

The subject is instructed to repeat successively increasing sequences of numbers forward (memory span, attention) and backward (working memory, executive function) on the "digit span" subtest of the Wechsler Memory Scale—Revised (WMS-R) [24].

### Regensburg-word-fluency-test

Semantic and phonemic verbal fluency will be tested using the “Regensburger Wortflüssigkeitstest” (RWT) [25] which also exists in parallel versions. In this test, the patient is asked to name as many words as possible that belong to a specific category (e.g. animals) within 1 min. For the phonemic word fluency subtest, the target is to name as many words as possible within 1 min that begin with a specific letter. This is a test for divergent thinking (executive function).

### Possible stress-related mediators and moderators

#### Anxiety and depression

The HADS-D [26] the German version of the Hospital Anxiety and Depression Scale by Zigmond and Snaith will be administered [27]. It is a standardized self-report screening instrument with two scales that capture anxiety and depression. Each scale is represented by seven items presented alternately. Each item is scored on a four-point Likert scale. The questions measure the expression of anxious and depressive symptoms referring to the last week. The items of the anxiety scale mainly refer to symptoms such as worry, apprehension, nervousness, and motor tension. The items of the depression scale focus on loss of motivation and interest, reduction of joy, and reduced drive. The questionnaire is well suited for recording reactive disorders in the physically ill. Another advantage is the quick completion time of approximately five minutes.

#### Self-assessment of cognitive failure

Study individuals will complete a validated German version of the Cognitive Failure Questionnaire for self-assessment (s-CFQ) [28]. It represents a procedure for self-assessment of the frequency of committed everyday errors, in the areas of perception, memory, and action regulation (executive functions). The CFQ has been used, revised, extended, and validated in numerous studies [28, 29].

### Stress experience

The Perceived Stress Scale [30] is used to assess subjective stress levels. With 10 items on a scale from “never” (0) to “very often” (4), the scale measures the occurrence of stress (feelings of being overtaxed, loss of control) in the last month. The German translation showed good internal consistency and construct validity [31].

We extended this questionnaire to include stress levels before and after testing prior to planned transplantation by the item “How stressed do you feel at the moment?” (1–10).

This allows us to measure the acute stress level at the beginning and at the end of the cognitive test and thus to investigate the difference in stress levels between the groups as well as the influence of the current stress level on cognitive functions.

### Hair cortisol

A hair sample is taken from the included individuals at each measurement time point for the determination of hair cortisol. Two to three thin hair strands are cut as close to the scalp as possible at the position of the posterior vertex. This protocol has been used successfully in studies for over 10 years and is well accepted by study participants [32]. The strands are first preserved and then sent to the laboratory of the Clinical Psychology of Adulthood at the University of Vienna (Prof. Dr. U. M. Nater) for hair cortisol analysis. For the determination of cortisol, commercial immunoassays with chemiluminescent detection (CLIA) from IBL, Hamburg, Germany, are used. For hair cortisol, high test–retest reliability as well as positive correlations with cortisol values from other media (e.g., saliva and urine), and with subjective stress measures are shown [33]. Hair cortisol levels of the first three centimeters from the scalp can be considered a measure of cumulative HPA axis activity over the previous three months [34].

In order to check the influence of steroids on the HPA axis, steroid doses are documented in all patients. Patients do not receive cortisone until transplantation. During transplantation, patients receive a cortisone shot of 1 g prednisolone on the day of transplantation. At the time of discharge from the hospital, the maintenance dose is 10 mg. The dose is usually reduced by 2.5 mg every 3 months and patients are no longer administered cortisone after 12 months. Furthermore, unscheduled intake of cortisone (e.g., during rejection episodes) is documented.

### Patient baseline data, comorbidity, laboratory parameters

In addition, baseline patient data, such as age, type of underlying disease, type and duration as well as the dose (*Kt/V*) of the dialysis therapy will be obtained. Comorbidities, that may have an impact on cognitive performance (such as previous stroke, coronary heart disease, hypertension diabetes mellitus, existing dementia or depression), will be documented. Furthermore, blood parameters such as hemoglobin, creatinine, calcium, phosphorus, albumin, triglycerides, cholesterine, urea, pH-value, CO_2_, and bicarbonate will be recorded. Current medication data will be obtained from the medical records and by self-report at each testing session. In addition, changes in medication are recorded. The examinations,were executed by trained and certified medical students.

### Statistical analysis

This is a within-group design with three measurement time points. We will investigate the proportion (percentage) of individuals that fulfill the above mentioned criteria at each given time point as estimates of prevalence of MCI and major neurocognitive impairment. Furthermore, we will conduct repeated-measures analysis of variance to investigate changes in cognitive performance over time, as well as the influence of different predictors. Cognitive performance of the individuals as a composite score and for each cognitive domain constitutes the dependent variables. The time of measurement and other control variables listed above represent the independent variables. A possible accumulation of the type I error is counteracted by an *α*-error correction (see below). The partial Eta-squared is calculated as the effect size, and the pairwise post-hoc comparisons are also corrected for multiple comparisons. The requirement of normal distribution is evaluated by the Kolmogorov–Smirnov test. Variance homogeneity is checked by Levene’s test. If the prerequisites for parametric statistics are not fulfilled, a distribution assumption-free covariance analysis according to the Quade model with rank-transformed variables is performed as an alternative.

Further questions are evaluated in terms of exploratory data analysis. Group differences are assessed using the t-test for independent samples or the Mann–Whitney-*U* test, depending on the scale level. Panels with discrete characteristics are analyzed with the *χ*^2^-test. Measures of correlation for discrete variables represent the contingency coefficients. For continuous characteristics, correlation measures according to Pearson (product-moment correlation) or Spearman (rank correlation) are used depending on the data level, in exceptional cases also according to Kendall (rank correlation without equidistance assumption).

The global significance level is set at *α* = 0.05. A false discovery rate (FDR) is calculated for *α*-error correction in multiple comparisons [35]. In the stepwise procedure, p-values are ranked in descending order; the null hypothesis is rejected if:

1 $$p(i) \le \frac{i}{m}\alpha$$

where *m* represents the number of *p* values and *i* the rank of the *p* value. Assuming *i* = 1, the FDR is equivalent to the Bonferroni correction.

### Sample size calculation

In order to investigate the prevalence of MCI or dementia, all individuals currently registered on the local transplant list, who are able to consent and agree to participate in the study, will be included. Currently, this list includes 142 individuals (75 individuals are in status T (transplantable), 67 individuals are in status NT (non-transplantable).

With regard to outcome measures, previous studies have shown highly significant improvements in some domains after transplantation [36], but have not reported effect sizes or statistical parameters that could be used to calculate effect sizes. Harciarek and colleagues [37, 38] report moderate to strong “time × comparison with healthy controls” interaction effects. We therefore conservatively expect to find low to medium effect sizes. For the detection of low to medium (*f* = 0.20) within-person effects with 3 measurement time points and 2 groups using a repeated-measures ANOVA as explained above, a power (1 – *β* (type II error probability) of 0.80 and a type-I error probability (*α*) of 0.05, a sample size of 42 is necessary [39, 40]. We therefore aim for a sample size of 50 persons for the follow-up measurements.

### Primary outcome measure

The primary outcome measure will be cognitive performance. (a) We will compare cognitive performance before and after kidney transplantation, up to 12 months after transplantation in a population of patients who did not yet receive kidney grafts before enrollment in the study (part A) and (b) we will also investigate the development of cognitive performance over a period of 12 months in a population of patients who already received kidney grafts before enrollment in the study (part B).

### Secondary outcome measures

As a secondary outcome, we will assess a distinct profile of cognitive function using standard neurocognitive tests. Furthermore, we will explore the impact and temporal development of a set of risk factors for deficits in cognitive performance. Second, we want to examine the extent to which cognitive impairment affects depression at all follow-up time points. Third, we want to assess how stressors negatively or positively affect cognitive performance. Fourth, we want to investigate to what degree cognitive performance affects the formation and development of donor-specific antibodies.

## Discussion

The purpose of this study is to assess the extent and development of cognitive impairment in kidney transplant individuals and to provide a clear profile of cognitive function using standardized neurocognitive tests. Furthermore, we aim to evaluate whether transplantation per se leads to an improvement in cognitive performance.

There is currently no standardized test battery for kidney transplant populations, so we selected different validated tests to evaluate the different domains. Through this, it will be possible to create a neurocognitive profile of the investigated population. The tests used in kidney transplant individuals [10, 11, 41, 42] (Brief Cognitive State Examination, MoCA und 3MS, Modified Mini-Mental State Examination), which can be found in literature, are only screening tests and may underestimate the extent of cognitive impairment. We use a more comprehensive neurocognitive test battery, which is then also able to establish a neurocognitive profile in individuals after kidney transplantation. A recently published study showed a high prevalence of cognitive impairment in dialysis individuals [5], examined by the CERAD Test battery [43].

Anemia, secondary hyperparathyroidism, dialysis disequilibrium and uremic toxins (UT) have been reported as major causes of cognitive impairment accompanied by chronic kidney disease [44], as well as dialysis duration [45]. We assume that these parameters improve after transplantation and want to investigate the effect of these parameters on cognitive function.

Depression in hemodialysis individuals is characterized as one of the most common psychological aspects regarding studies on individuals with kidney failure [46]. To evaluate the frequency of depression and its effect on cognitive performance the HADS-D [26] Test will be performed. We also hypothesize that transplanted individuals suffering from depression display significantly higher cognitive impairment than transplanted individuals without depression. In one of our previous investigations, depression was significantly associated with a lower level of cognition [5]. Other studies have also found similar decline in cognition with the presence of depression [45, 47, 48]. This can be explained by the effects of symptoms of depression on domains of cognition like executive functioning and processing speed [48, 49]. A recent meta-analysis also shows that subjective stress influences the development of cognitive impairment [50].

A likely mediator of the association between stress and cognitive performance, but also between depression and cognitive performance, is HPA axis activity, which is commonly assessed via its end-product cortisol. Excess cortisol has been found to have damaging effects on the limbic system, which leads to impairment of learning mechanisms [51]. Some studies also suggest that higher cortisol levels are associated with slower processing speed in persons suffering from depression [52, 53]. In line with this, one study found a negative association between hair cortisol and cognitive performance after stroke [54]. Hair cortisol as a marker of HPA axis activity is of special interest because it represents the cumulative HPA axis activity of the months before the time point of measurement [33] (in our case, before kidney transplant). However, we are not aware of any study that has investigated associations of subjective stress or hair cortisol, or their interaction with depression, in kidney transplant individuals.

Individuals who received kidneys from a deceased donor as well individuals who received living kidney donation will be investigated. In order to detect an effect of transplantation, testing will be performed close to the time of transplantation. Neurocognitive testing is performed in living kidney donor recipients within 14 days prior to planned transplantation. In individuals receiving postmortem donation, prior scheduling of neurocognitive testing is not possible. After individuals are placed on the waiting list, the waiting period for a deceased donor renal transplant usually ranges between 6 to 8 years in Germany [55] and around 4 years in the Eurotransplant region [56], making testing at listing impractical. Therefore, we decided to perform testing when we admit individuals for kidney transplantation during dialysis prior to transplantation. It has been shown that the test results are not affected by dialysis [57].

Despite vigorous planning, the present investigation certainly contains some limitations. The test environment may not be optimal for the individuals who will receive the kidney from a deceased donor (patient might be more nervous than usual). It would have been more ideal to test those individuals 1 or 2 weeks before transplantation, but this is not possible, as it is not known when these individuals will receive their transplant offers. It seems possible that HADS responses may be influenced by the positive news of the transplantation. Here, however, it is likely that the influence might be more pronounced in individuals for whom transplantation is not planned (deceased donor kidney transplantation) than in those for whom transplantation is planned (living donation).

The results of our study could have potentially important implications for the prevention and treatment of cognitive impairment in kidney transplant individuals. By increasing our knowledge of the neurocognitive profile and assigning the corresponding deficits, it might be possible to create an individualized training program to positively impact cognitive deficits in these individuals.

### Trial status

The study is currently enrolling individuals. The local human research ethics committee of the Justus-Liebig-University of Giessen (AZ 195/20) approved this study. Recruitment started in Jan 2021 and is expected to be completed in December 2022. The study was registered with the German clinical Trials register under the number DRKS00029164.

## Data Availability

The datasets used or analyzed during the current study are available from the corresponding author on reasonable request.

## Abbreviations

sCFQ: Questionnaire for self-assessment
CKD: Chronic kidney disease
ESRD: End-stage renal disease
HADS-D: The German version of the Hospital Anxiety and Depression Scale
HPA: Hypothalamic-pituitary-adrenocortical
MCI: Mild cognitive impairment
MMSE: Mini-mental-state-Examination
NAsKiT: Neuropsychological Assessment of Cognitive Impairment in Kidney Transplantation
ROCFT: The Rey-Osterrieth Complex Figure Test
RWT: The “Regensburger Wortflüssigkeitstest
TMT: Trail Mark Test
VLMT: The “verbaler Lern- und Merkfähigkeitstest

## Code availability

No new code was produced in this study.
